# Analysis of association between eating behaviours and childhood obesity among pre-school children: A cross-sectional study

**DOI:** 10.3389/fped.2022.1073711

**Published:** 2023-01-06

**Authors:** Yi-Xin Wu, Hai-Ling Fan, Jin Dai, Hui-Lan Wu, Jing-Yun Yang, Yun Wang, Tao-Hsin Tung, Li-Zhen Wang, Mei-Xian Zhang

**Affiliations:** ^1^ Department of Paediatrics, Taizhou Hospital of Zhejiang Province Affiliated to Wenzhou Medical University, Linhai, China; ^2^ Department of Paediatrics, Taizhou Hospital of Zhejiang Province Affiliated to Wenzhou Medical University, Luqiao Hospital, Taizhou Enze Medical Center, Luqiao, China; ^3^ Evidence-based Medicine Center, Taizhou Hospital of Zhejiang Province Affiliated to Wenzhou Medical University, Linhai, China

**Keywords:** pre-school children, obesity, eating behaviours, children's eating behaviour questionnaire, weight status

## Abstract

**Background:**

Childhood obesity is a worldwide critical health concern. We aimed to clarify whether eating behaviours increased the risk of childhood obesity.

**Methods:**

We recruited 2,049 pre-school children aged 3–6 years between 1 December 2021 and 31 January 2022 in Taizhou, China. Children's weight status was classified according to the International Obesity Task Force criteria, and their eating behaviours were evaluated using the Children's Eating Behaviour Questionnaire. Correlation analyses, linear regressions, and one-way ANCOVA. were performed to analyse the association between children's eating behaviours and weight status.

**Results:**

In ‘Food Avoidant' subscales, the scores of satiety responsiveness (*P* < 0.001) and slowness in eating (*P* = 0.001) were negatively associated with body mass index z score among pre-school children of both sexes. In ‘Food Approach’ subscales, the score of enjoyment of food was positively associated with body mass index z score in both boys (*P* = 0.007) and girls (*P* = 0.035), but the association of scores of food responsiveness with body mass index z score was found only in girls (*P* = 0.001).

**Conclusion:**

Our results supported that pre-school children with low scores in ‘Food Avoidant’ subscales and high scores in ‘Food Approach’ scales were more likely to become obese.

## Introduction

Childhood obesity is a worldwide critical health concern, the prevalence of which has increased at an alarming rate (1). It is also a major public health issue in China, with the latest prevalence estimates for 2015–2019 being 6.8% overweight and 3.6% obese pre-school children; along with 11.1% overweight and 7.9% obese school children and adolescents aged 6–17 years (2). A study has reported the persistence of obesity from childhood to adulthood (3). Childhood obesity is also considered a risk factor for various chronic diseases, including diabetes, cardiovascular diseases, and cancers in adulthood (4–6).

Emerging evidence suggests that childhood obesity can be attributed to a variety of components such as parental weight, socioeconomic factors, and eating behaviours (7, 8). Several studies confirmed that specific eating behaviours could be considered a susceptible factor for childhood obesity in China and other countries (9–13). There is strong evidence that children's eating behaviours are mostly formed in the pre-school age, and early intervention at this age to form healthy eating habits is considered to be one of the best ways to slow down the progression of childhood obesity (8, 14, 15). A cross-sectional study conducted in the mega cities of China demonstrated that school environment and policies helped children to develop good eating habits and also reduced the risk of childhood obesity (16). Therefore, it is necessary to identify eating behaviours associated with childhood obesity and cultivate healthy eating habits when a child is in the pre-school stage. However, the association of eating behaviours with childhood obesity has been reported to vary across ages, parents and cultural environments (17–19). Majority of similar research population samples are mainly from Europe, North America, and mega cities of China (14, 20). Taizhou, a small city in East China, has a unique economic and cultural background, which is different from the major cities of China and developed Western ­countries. The association between eating behaviours and childhood obesity among pre-school children has seldom been studied in Taizhou. Thus, this study aimed to clarify whether eating behaviours could increase the risk of childhood obesity in the children from Taizhou. We hypothesized that there might be a relationship between eating behaviours and childhood obesity. Pre-school children with low scores in ‘Food Avoidant’ subscales and high scores in ‘Food Approach’ scales would be more likely to become obese. Understanding the association between eating behaviours and childhood obesity may help paediatricians formulate screening and management strategies for pre-schoolers with obesity.

## Materials and methods

### Study participants

We recruited pre-school children (*N* = 2049), aged 3–6 years old from local kindergartens between 1 December 2021 and 31 January 2022 in Taizhou. Exclusion criteria for the original study included children younger than 3 years of age and older than 7 years (*N* = 29), repeating cases (*N* = 10), and outliers (*N* = 75).

This study was approved by the Ethics Committee of Taizhou Hospital of Zhejiang Province (approval number: K20220123) in China. Both children and guardians provided informed consent to participate in this study and cooperated voluntarily. All procedures were performed in accordance with the guidelines of our institutional ethics committee and complied with the tenets of the Declaration of Helsinki.

### Measures

We designed a questionnaire including the Children's Eating Behaviour Questionnaire (CEBQ) and added additional questions regarding children's birth date, sex, living environment, current weight and height, number of siblings, along with parents' weight, height, occupation, level of education, and family's yearly income. The CEBQ has been widely used to evaluate the eating behaviours of children aged 2–13 years and has achieved good internal consistency, test-retest reliability, and reasonable construct validity (21, 22). The CEBQ comprises 35 items and covers ‘Food Avoidant’ and ‘Food Approach’ subscales. The ‘Food Avoidant’ subscales consists of satiety responsiveness [SR], slowness in eating [SE], emotional under-eating [EUE], and food fussiness [FF]. While, the ‘Food Approach’ subscales include emotional over-eating [EOE], food responsiveness [FR], enjoyment of food [EF], and desire to drink [DD]. Please ignore if not applicable (22). Parents were asked to rate their children's eating behaviour in the past three months on a five-point Likert scale (never, rarely, sometimes, often, always; 1–5).

This was a self-administered questionnaire distributed electronically to parents *via* a platform named ‘wenjuanxing’. Parents voluntarily completed and submitted the questionnaires. All the questions were compulsory and could only be submitted if the entire questionnaire was completed. Children's weight and height were reported by parents, while parents' weight and height were self-reported.

Body mass index (BMI) for all the participants was calculated as BMI = weight (kg)/height(m)^2^. Based on the International Obesity Task Force (IOTF), each child's BMI was adjusted for age and sex and converted to a standardised z score. Children were diagnosed as underweight, normal weight, and overweight/obese based on these scores (23). Parents' weight status was classified as underweight (BMI < 18.5), normal weight (18.5 ≤ BMI < 25), and overweight/obesity (BMI ≥ 30), based on the international cut-off points (24).

### Statistical analysis

Continuous variables with approximate normal distribution were assessed using histograms, while categorical variables were presented as means (standard deviation [SD]) and frequencies (percentages). Values (children's body mass index z score [BMIZ] and CEBQ scores) that deviated from the mean by more than three SDs were considered outliers. Cronbach's alphas were calculated to assess internal reliability of the questionnaire. Chi-square test was used to analyse differences in participants' characteristics according to the children's weight status.

Association of the scores of eight subscales of CEBQ with children's BMIZ were assessed using correlation analyses, linear regressions, and one-way ANCOVA. First, we calculated Pearson correlation coefficient to understand the linear relationship between eight subscales of CEBQ and children's BMIZ. Then, in order to quantify this relationship, we ran a series of regressions using children's BMIZ as the outcome, eight subscales of CEBQ as predictors, and other information (e.g., children's age, children's living environment, parents' weight status, parents' level of education, parents' occupation, family's yearly income, number of siblings) as covariates. Finally, we ran one-way ANCOVA after adjusting for covariates, along with *post hoc* Bonferroni analysis, to visually demonstrate differences between weight status. All these analyses were conducted by sex.

All statistical analyses were performed using IBM SPSS Statistics 23.0. (NY, United States) Statistical significance was set at *P* < 0.05 (two-sided).

## Results

### Characteristics by children's weight status

Table 1 presents the children's demographic and socioeconomic characteristics. A total of 1935 children aged 3–6 years with a mean BMIZ of −0.08 ± 1.85 were analysed in this study, of whom 1,030 were boys and 905 were girls. More than half of the children had normal weight (59.4%), while a low proportion of them were overweight or obese (18.0%). The remaining children were underweight (22.6%). Distribution of weight status among mothers was similar to that of the children: 74.2% mothers were classified as having normal weight, 16.0% as overweight/obese, and 10.2% as underweight. Compared to the children's weight status, fathers showed larger proportion of being overweight/obese (43.5%) and lower proportion of being underweight (2.0%). The mean BMI for fathers and mothers was 25.00 ± 4.75 kg/m^2^ and 22.73 ± 5.43 kg/m^2^, respectively. Parental BMI levels highly correlated with children's BMI, and children who were raised by obese parents had a correspondingly higher obesity rate (*P* < 0.001). Furthermore, the odds of childhood obesity increased with age (*P* = 0.045) in these individuals. Additionally, it was observed that children living in families with higher annual incomes or children raised by more educated parents had lower rates of being overweight and obese. In general, apart from children's sex and fathers' occupation, other parental and socioeconomic characteristics significantly differed across children's weight status (*P* < 0.05).

**Table 1:** Differences in participants’ characteristics by children's weight status (*n* (%), *N *= 1935).

Variables	Total (*N* = 1935)	Underweight (*N* = 438)	Normal weight (*N* = 1149)	Overweight/Obesity (*N* = 348)	*p*-Value
Children's age (years), *n* (%)					0.045
3 years	475 (24.5)	124 (26.1)	271 (57.1)	80 (16.8)	
4 years	633 (32.7)	150 (23.7)	386 (61.0)	97 (15.3)	
5 years	637 (32.9)	125 (19.6)	378 (59.3)	134 (21.0)	
6 years	190 (9.8)	39 (20.5)	114 (60.0)	37 (19.5)	
Children's gender, *n* (%)					0.159
Boy	1,030 (53.2)	224 (21.7)	632 (61.4)	174 (16.9)	
Girl	905 (46.8)	214 (23.6)	517 (57.1)	174 (19.2)	
Children's living environment, *n* (%)					0.010
Urban	1,230 (63.6)	273 (22.2)	758 (61.6)	199 (16.2)	
Rural	705 (36.4)	165 (23.4)	391 (55.5)	149 (21.1)	
Father's weight status, *n* (%)					<0.001
Underweight	38 (2.0)	14 (36.8)	22 (57.9)	2 (5.3)	
Normal	1,056 (54.6)	259 (24.5)	639 (60.5)	158 (15.0)	
Overweight/Obesity	841 (43.5)	117 (18.5)	389 (61.6)	188 (22.4)	
Mother's weight status, *n* (%)					<0.001
Underweight	198 (10.2)	64 (32.3)	106 (53.5)	28 (14.1)	
Normal	1,427 (73.7)	304 (21.3)	887 (62.2)	236 (16.5)	
Overweight/Obesity	310 (16.0)	37 (20.7)	98 (54.7)	84 (27.1)	
Father's educational level, *n* (%)					0.019
Junior high school or below	301 (15.6)	77 (25.6)	154 (51.2)	70 (23.3)	
High school	532 (27.5)	117 (22.0)	318 (59.8)	97 (18.2)	
University or above	1,102 (57.0)	244 (22.1)	677 (61.4)	181 (16.4)	
Mother's educational level, *n* (%)					<0.001
Junior high school or below	276 (14.3)	64 (23.2)	136 (49.3)	76 (27.5)	
High school	424 (21.9)	111 (26.2)	237 (55.9)	76 (17.9)	
University or above	1,235 (63.8)	263 (21.3)	776 (62.8)	196 (15.9)	
Father's occupations, *n* (%)					0.128
Brain work	1,595 (82.4)	356 (22.3)	961 (60.3)	278 (17.4)	
Physical work	133 (6.9)	28 (21.1)	71 (53.4)	34 (25.6)	
Other occupation	207 (10.7)	54 (26.1)	117 (56.5)	36 (17.4)	
Mother's occupations, *n* (%)					0.042
Brain work	1,456 (75.2)	336 (23.1)	880 (60.4)	240 (16.5)	
Physical work	101 (5.2)	18 (17.8)	59 (58.4)	24 (23.8)	
Other occupation	378 (19.5)	84 (22.2)	210 (55.6)	84 (22.2)	
Yearly family income, *n* (%)					0.011
120,000 or below	410 (21.2)	90 (22.0)	224 (54.6)	96 (23.4)	
120,000-500,000	1,245 (64.3)	286 (23.0)	745 (59.8)	214 (17.2)	
500,000 or above	280 (14.5)	62 (22.1)	180 (64.3)	38 (13.6)	
Number of siblings, *n* (%)					0.019
0	813 ( 42.0)	175 (21.5)	511 (62.9)	127 (15.6)	
1 or above	1,122 (58.0)	263 (23.4)	638 (56.9)	221 (19.7)	

### Reliability of CEBQ

Table 2 presents Cronbach's alphas for the CEBQ. Coefficients for each subscale of the CEBQ ranged between 0.68 and 0.81. The overall Cronbach's coefficient alpha of this questionnaire was 0.80, which indicated acceptable internal consistency.

**Table 2:** Reliability of CEBQ (*n* = 1935).

Subscale	Cronbach's coefficient α
Satiety responsiveness	0.70
Slowness in eating	0.81
Food fussiness	0.68
Emotional under-eating	0.70
Food responsiveness	0.77
Enjoyment of food	0.79
Desire to drink	0.68
Emotional over-eating	0.73
Overall	0.80

### Association between the scores of eight subscales of CEBQ and children's BMIZ

Table 3 details Pearson correlation coefficient between the CEBQ subscales and children's BMIZ. This study demonstrated that the ‘Food Approach’ subscales were positively related with children's BMIZ, while the ‘Food Avoidant’ subscales were negatively related with children's BMIZ. Significant correlations were found in terms of SR (*P* = 0.002), SE (*P* < 0.001), and EF (0.032) among boys. In girls, significant correlations were found in the context of SR (*P* < 0.001), SE (*P* < 0.001), FR (*P* = 0.001), and EF (*P* = 0.034).

**Table 3:** Pearson's correlations between the CEBQ subscales and children's BMIZ (*N* = 1935).

Subscale	Boy		Girl	
	r	*P*-value	r	*P*-value
‘Food Avoidant’ subscale				
Satiety responsiveness	−0.096	0.002	−0.126	<0.001
Slowness in eating	−0.111	<0.001	−0.118	<0.001
Food fussiness	−0.025	0.414	−0.007	0.827
Emotional under-eating	−0.041	0.184	−0.032	0.329
‘Food Approach’ subscale				
Food responsiveness	0.025	0.422	0.106	0.001
Enjoyment of food	0.055	0.032	0.071	0.034
Desire to drink	0.035	0.268	0.003	0.930
Emotional over-eating	0.033	0.287	0.045	0.173

Table 4 presents the results of linear regression analyses for children's BMIZ on CEBQ subscales. Eating behaviours were found to be associated with children's BMIZ in correlation analyses. To quantify this association, children's BMIZ were regressed on scores for each CEBQ subscale separately after controlling for the covariates. The results showed that SR (boys: *β* −0.090∼-0.012, *P* = 0.010; girls: *β* −0.112∼-0.029, *P* = 0.001) and SE (boys: *β* −0.095∼-0.019, *P* = 0.003; girls: *β* −0.105∼-0.022, *P* = 0.003) in ‘Food Avoidant’ scales had significant negative associations with children's BMIZ; and EF (boys: *β* 0.003∼0.077, *P* = 0.045; girls: *β* 0.010∼0.098, *P* = 0.010) in ‘Food Approach’ Scales had significant positive associations with BMIZ amongst both boys and girls. In addition to the above subscales being statistically significant, FR (*β* 0.035∼0.110, *P* < 0.001) was also found to positively associated with BMIZ among girls.

**Table 4:** Linear regression analyses for children's BMIZ on CEBQ subscales (*N* = 1935).

Subscale	Boy		Girl	
	*β* coefficient (95% CI)	*P*-value	β coefficient (95% CI)	*P*-value
‘Food Avoidant’ subscale				
Satiety responsiveness	−0.051 (-0.090∼-0.012)	0.010	-0.070 (-0.112∼-0.029)	0.001
Slowness in eating	-0.057 (-0.095∼-0.019)	0.003	-0.064 (-0.105∼-0.022)	0.003
Food fussiness	-0.011 (-0.043∼0.021)	0.494	0.008 (-0.027∼0.443)	0.653
Emotional under-eating	-0.023 (-0.064∼0.019)	0.280	-0.009 (-0.059∼0.040)	0.718
‘Food Approach’ subscale				
Food responsiveness	-0.023 (-0.014∼0.060)	0.219	0.073 (0.035∼0.110)	<0.001
Enjoyment of food	0.037 (0.003∼0.077)	0.045	0.054 (0.010∼0.098)	0.015
Desire to drink	0.034 (-0.021∼0.088)	0.224	0.016 (-0.045∼0.077)	0.604
Emotional over-eating	0.028 (-0.016∼0.072)	0.217	0.042 (-0.007∼0.091)	0.096

Covariates: children's age, children's living environment, parents’ weight status, parents’ educational level, parents’ occupation, yearly family income, number of siblings.

**Figure 1:**
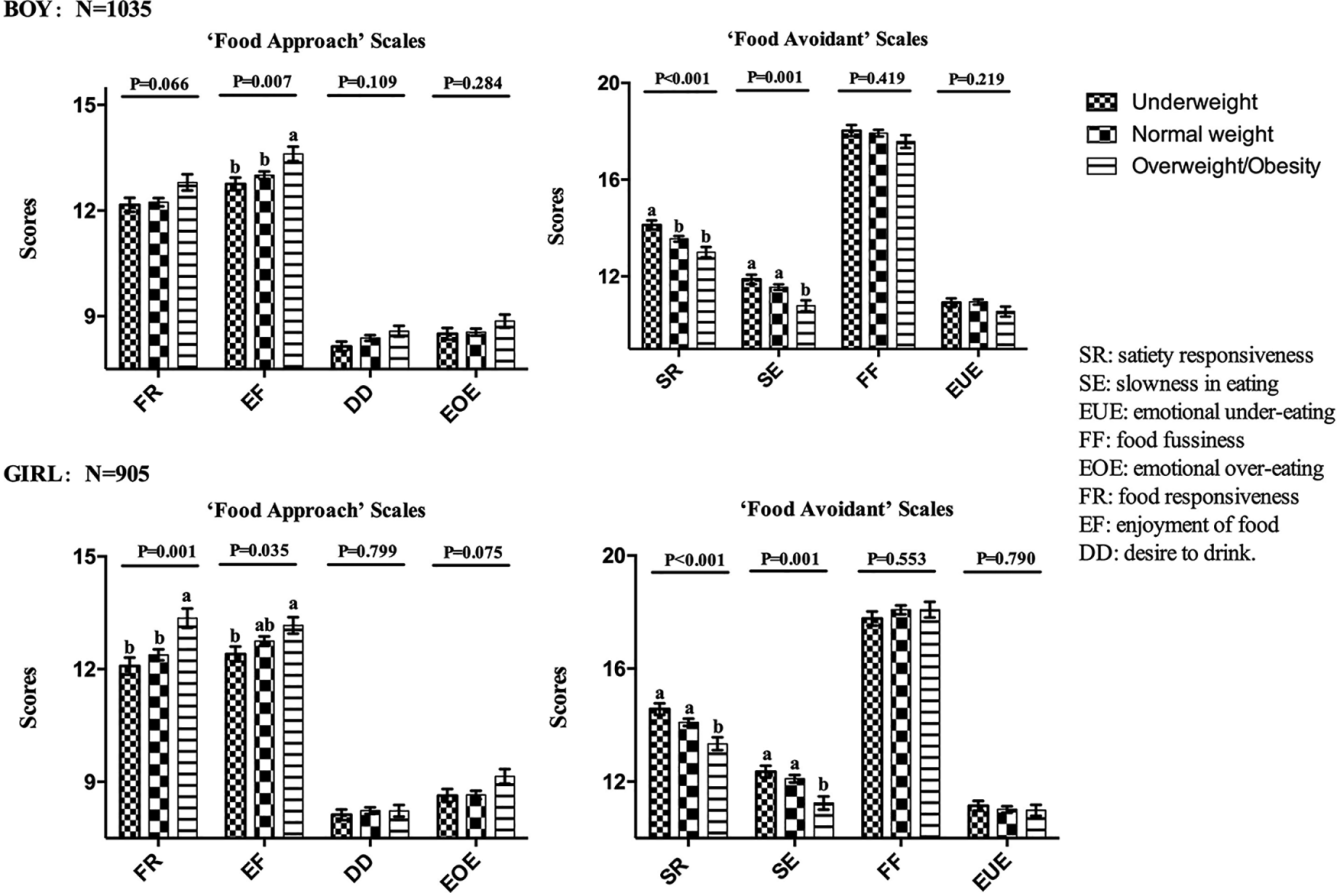
**CEBQ scores for children with different weight status (*N* = 1935)**. The general linear model was adjusted for following covariates: children's age, children's living environment, parents’ weight status, parents’ educational level, parents’ occupation, yearly family income, number of siblings. SR: satiety responsiveness. SE: slowness in eating, EUE: emotional under-eating, FF: food fussiness, EOE: emotional over-eating, FR: food responsiveness, EF: enjoyment of food, DD: desire to drink. ^a,b^Mean values within each subscale with unlike superscript letters were significantly different: *P* < 0.05.

Figure 1 details CEBQ scores for children with different weight status stratified by sex. The above findings showed that eating behaviours were strongly related to children's BMIZ. Therefore, to visually demonstrate differences between weight status, we used a general linear model after adjusting for covariates. In boys, significant differences were observed in the scores of SR (*P* < 0.001) and SE (*P* = 0.001) in ‘Food Avoidant’ scales, and EF (*P* = 0.007) in ‘Food Approach’ Scales among the three groups of children with weight status. Girls were almost identical to boys in ‘Food Avoidant’ scales (SR: *P* < 0.001; SE: *P* = 0.001), but slightly different in ‘Food Approach’ Scales. Significant differences were noted in EF (*P* = 0.035) subscales, and FR (*P* = 0.001) between the two sexes.

## Discussion

This study detailed the prevalence of obesity among pre-school children in Taizhou. The childhood overweight and obesity rate was 18.0% in Taizhou, which was higher than the 2015–2019 average level across China (10.4%) (2). This result emphasizes the need of urgent attention towards childhood obesity from paediatricians and parents in Taizhou.

Our study identified an association between eating behaviours and childhood obesity among pre-school children in Taizhou, which has also been confirmed by previous studies conducted in other countries (10–13, 25, 26). Furthermore, we found the general trend that ‘Food Approach’ subscales were positively associated with children's BMIZ, while the ‘Food Avoidant’ subscales were negatively associated with children's BMIZ. This finding was in concordance with previous studies (10–13, 25, 26). However, the association of various subscales of the CEBQ with childhood obesity remains controversial.

‘Satiety responsiveness’ subscale reflects the ability to regulate the amount of food consumed according to internal satiety cues. ‘Slowness in eating’ subscale is intended to reflect gradual reduction of interest in a meal (22). These two subscales were shown to be negatively associated with BMIZ in this study. Children in the overweight/obesity group had the lowest scores of these subscales in comparison to the other two groups. Similar to the findings of this study, both these subscales have been reported to be strongly associated with childhood obesity in previous studies (10–13, 26). Therefore, we can assume that eating behaviours represented by these two subscales highly correlate with childhood obesity.

‘Enjoyment of food’ subscale represents a general interest in food (22). Overweight/obese children had higher scores on this subscale compared to other groups, and it can be considered that this subscale highly correlates with childhood obesity. Similar results have been obtained in studies in Netherlands (10), Chile (13), Canada (11), Portugal (12), and Saudi (26). Therefore, paediatricians need to monitor children for similar eating behaviours so that they can identify children who are at risk of being overweight or obese at the earliest.

‘Food responsiveness’ subscale intends to measure eating in response to external food cues (22). It is worth mentioning that this is the only subscale in which different results were obtained for boys and girls in our study. In girls, the FR subscales gained the highest scores in overweight/obesity group. However, we did not observe any association between the two amongst boys. The association between FR and children's weight status has been reported in previous studies (10–13, 26). While, only few studies have focused on examining differences in FR between boys and girls. A study (13) conducted in Chile examined whether sex differences had an effect on the association between FR and childhood obesity and concluded that the association of FR with childhood obesity was independent of sex. In our study, it was observed that although the association of FR with children's weight status was not statistically significant among boys (*P* = 0.066), a general trend was clearly evident that BMIZ increased with increase in FR score. Therefore, we can presume that both boys and girls who score high on FR subscale may have a higher likelihood of developing obesity compared to others in the future.

‘Food fussiness’ subscale reflects a lack of interest in food and reluctance to try new foods, resulting in insufficient food variety (22). Scores on this subscale did not vary according to children's weight status in our study. The association of FF with children's weight status remains unclear according to the previous studies. For example, surveys conducted in Portugal (12) and Canada (11) suggested that FF may lead to weight loss in children, whereas surveys conducted in Chile (13), Netherlands (10), and Saudi (26) suggested that FF was not associated with children's weight status. It is not surprising that the association of FF with children's weight status has diverged in several studies. FF leads to reduction in dietary diversity, which is associated with poor diet quality (27). However, even if a child's diet quality decreases due to FF, the child is at no risk of being underweight, as long as he or she consumes adequate amounts of food.

‘Emotional overeating and undereating’ subscales are characterised by increased or decreased eating in response to negative emotions, such as anger and anxiety (22). These two subscales were not found to be associated with childhood obesity in our study. The association between emotional eating behaviours and obesity is more validated in school-aged children, whereas the association between the two remains in question among pre-school children (25, 28, 29). A systematic review (28) mentioned that emotion control training may be an effective behaviour change technique for preventing or managing childhood obesity. However, this study reported the findings of children aged 2­–18 years, and it is not known if these findings can be effectively applied to pre-school children. Therefore, further research is needed to clarify the association between emotion-related eating behaviours and obesity.

‘Desire to drink’ subscale was developed to detect increased cravings for beverages, especially sugar-sweetened beverages. It has been associated with a preference for drinking sugar-sweetened beverages (22). DD did not correlate with children's weight status in this study. The association of DD with weight status has not been conclusively established in previous studies. A cross-sectional study conducted in Canada (11) reported no association, whereas a similar study conducted in Portugal (12) showed significant associations between DD and weight status. We believe that this situation has to be attributed to the different dietary habits in each country. Our study showed that childhood obesity in pre-school children was not associated with DD because Chinese children are less dependent on beverages compared to children of other countries. Sugary beverages, such as soft drinks, have long been proven to be high-energy refreshments, which are closely associated with childhood obesity (30). Although the present study did not suggest that obesity in pre-school children in Taizhou was associated with DD, children with higher scores on this subscale may be at a higher risk of being overweight or obese.

Pre-school children who scored higher on FR and EF and lower on SE and SR have been reported to have increased appetite (31, 32). For example, they increase food intake when exposed to food cues, show higher levels of food consumption in the absence of hunger, and do not show deceleration pattern of intake during eating (31, 32). Previous studies have suggested that appetite characteristics were significantly related to changes in adiposity (32). Therefore, it can be considered that these children would be at a higher risk of developing obesity with more significant fat accumulation in comparison to others.

The strengths of our study include collection of large number of samples with different family incomes across urban and rural areas, suggestive of good representation of the population, and use of a reliable and effective questionnaire. However, this study had some limitations. First, this was a cross-sectional study, rather than a cohort study, which did not confirm the causal inferences, but provided a direction for future cohort studies. Second, children's weight and height were provided by the parents; therefore, there is a certain chance that wrong data could have been provided. Third, the relatively low response rate may have led to bias in the study results.

This study suggests that if paediatricians encounter parents who come for consultation regarding obesity-related issues in their clinics, they can carefully enquire about the medical history of children's eating behaviours to determine whether children have unhealthy eating behaviours related to obesity, as mentioned in the above results. If this kind of eating behaviour exists in the daily lives of children, paediatricians should consider the possibility that the child may develop obesity. They must intervene in the child's eating behaviour at an early stage to help the child form healthy eating behaviours and slow down the progression of obesity.

In conclusion, we found that the prevalence of obesity among pre-school children in Taizhou was comparatively higher than the average Chinese level. Meanwhile our results supported the finding that pre-school children with low scores in ‘Food Avoidant’ subscales (boys and girls: SR and SE) and high scores in ‘Food Approach’ scales (boys: EF, girls: EF and FR) were more likely to suffer from obesity.

## Data Availability

The raw data supporting the conclusions of this article will be made available by the authors, without undue reservation.
